# Design and Evaluation of Autophagy-Inducing Particles for the Treatment of Abnormal Lipid Accumulation

**DOI:** 10.3390/pharmaceutics14071379

**Published:** 2022-06-29

**Authors:** Stavroula Zagkou, Valentine Marais, Narimane Zeghoudi, Edouard Le Guillou, Eeva-Liisa Eskelinen, Ganna Panasyuk, Bernard Verrier, Charlotte Primard

**Affiliations:** 1 Adjuvatis, 60 Avenue Rockefeller, 69008 Lyon, France; valentine.marais@adjuvatis.com (V.M.); narimane.zhegoudi@adjuvatis.com (N.Z.); 2 Laboratory of Tissue Biology and Therapeutic Engineering, CNRS UMR 5305, Université Claude Bernard Lyon 1, CEDEX 7, 69367 Lyon, France; 3 INSERM UMR-S1151, CNRS UMR-S8253, Institut Necker Enfants Malades, Université Paris Cité, 75015 Paris, France; edouard.le-guillou@inserm.fr (E.L.G.); ganna.panasyuk@inserm.fr (G.P.); 4 Institute of Biomedicine, University of Turku, 20520 Turku, Finland; eeva-liisa.eskelinen@utu.fi

**Keywords:** autophagy, polylactic acid, polymeric nanoparticles, liver targeting, Tat-Beclin peptide, non-alcoholic fatty liver disease

## Abstract

Autophagy is a fundamental housekeeping process by which cells degrade their components to maintain homeostasis. Defects in autophagy have been associated with aging, neurodegeneration and metabolic diseases. Non-alcoholic fatty liver diseases (NAFLDs) are characterized by hepatic fat accumulation with or without inflammation. No treatment for NAFLDs is currently available, but autophagy induction has been proposed as a promising therapeutic strategy. Here, we aimed to design autophagy-inducing particles, using the autophagy-inducing peptide (Tat-Beclin), and achieve liver targeting in vivo, taking NAFLD as a model disease. Polylactic acid (PLA) particles were prepared by nanoprecipitation without any surfactant, followed by surface peptide adsorption. The ability of Tat-Beclin nanoparticles (NP T-B) to modulate autophagy and to decrease intracellular lipid was evaluated in vitro by LC3 immunoblot and using a cellular model of steatosis, respectively. The intracellular localization of particles was evaluated by transmission electron microscopy (TEM). Finally, biodistribution of fluorescent NP T-B was evaluated in vivo using tomography in normal and obese mice. The results showed that NP T-B induce autophagy with a long-lasting and enhanced effect compared to the soluble peptide, and at a ten times lower dose. Intracellular lipid also decreased in a cellular model of NAFLD after treatment with T-B and NP T-B under the same dose conditions. Ultrastructural studies revealed that NP T-B are internalized and located in endosomal, endolysosomal and autolysosomal compartments, while in healthy and obese mice, NP T-B could accumulate for several days in the liver. Given the beneficial effects of autophagy-inducing particles in vitro, and their capacity to target the liver of normal and obese mice, NP T-B could be a promising therapeutic tool for NAFLDs, warranting further in vivo investigation.

## 1. Introduction

Autophagy is a fundamental catabolic house-cleaning process by which cells degrade their cytoplasmic cargo to maintain homeostasis. It is conserved in all cells and across all eukaryotic species [1]. Mammalian macroautophagy (autophagy) starts by the formation of cup-shaped structures called phagophores which elongate and engulf portions of the cytoplasm. Phagophores will eventually become double-membraned autophagosomes which fuse with lysosomes, resulting in cargo degradation by hydrolases in the autolysosomes [2]. The degradation products, including amino acids, lipids, carbohydrates and nucleic acids, are recycled back to the cytoplasm where they serve as energy sources or as substrates for biosynthetic pathways [2]. Autophagy-related (ATG) proteins regulate the key steps in autophagosome formation, including initiation, nucleation, elongation and lysosome fusion. The major components of the autophagic machinery are: the Ser/Thr protein kinase ULK1∕2 complex, the lipid kinase class 3 phosphatidylinositol 3-kinase (PI3K) complexes, the ATG9 trafficking system and the ubiquitin-like conjugation systems, ATG12-ATG5 and ATG8 proteins, including MAP1LC3/LC3 (microtubule-associated protein 1 light chain 3 beta). LC3 proteins are conjugated to the lipid phosphatidylethanolamine, yielding LC3-II, which can be separated from the unlipidated LC3-I in sodium dodecyl sulfate-polyacrylamide gel electrophoresis (SDS-PAGE). LC3-II is the only protein marker that is reliably associated with completed autophagosomes [3]. Macroautophagy can be either non-selective or selective; the former involves random sequestration of cytoplasmic material by autophagosomes, for example under nutrient starvation conditions [4]. In contrast, selective autophagy occurs under nutrient-rich conditions. Depending on the cargo to be degraded and the molecular machinery involved, different types of selective autophagy have been identified, for example xenophagy (selective degradation of intracellular pathogens) [5], mitophagy (removal of damaged mitochondria) [6] and lipophagy (degradation of lipid droplets) [7].

Dysregulation of autophagy has been associated with various human diseases, most notably neurodegeneration [8], cancer [9] and metabolic diseases [10]. Among the latter, non-alcoholic fatty liver (NAFL) has gained epidemic proportions and its progression to steatohepatitis (NASH) is a major health problem [11]. Approximately one in four people suffer from non-alcoholic fatty liver diseases (NAFLDs) worldwide, and in the United States of America NAFL and NASH are affecting 30% and 5% of the population, respectively [12]. NAFL and NASH are characterized by hyperlipidemia, inflammation, ballooning and apoptotic hepatocellular injury, and are often accompanied by insulin resistance and obesity, which collectively comprise the metabolic syndrome. Furthermore, NASH may gradually progress to cirrhosis and hepatocellular carcinoma, with patients requiring a liver transplant later in life [13]. In the absence of clinically approved pharmacological treatment, the first line of treatment for NAFLDs includes lifestyle modification consisting of weight loss and exercise [14], yet patient compliance with these approaches is low. Recently, a novel role of autophagy in degrading lipid droplets (lipophagy) [7] has been identified, which led to the hypothesis that autophagy induction can be beneficial in the context of NAFLDs. In recent studies, substances that induce autophagy, including rapamycin, a novel TFEB inducer, herbal medicines and FDA-approved drugs such as metformin, ezetimibe, irbesartan, pioglitazone and exenatide, have shown to ameliorate NAFLDs in different animal models [15]. Yet, in most of these cases, the observed therapeutic effects cannot be exclusively attributed to autophagy. Importantly, the context-dependent role of autophagy hampers the translation of autophagy-inducing therapeutics for NAFLDs [16]. For example, in the context of NAFLDs and obesity in humans, autophagy is activated in the white adipose tissue [17], but decreased in the liver [18]. Elucidating how tissue-specific autophagy contributes to disease, together with the development of tools to selectively modulate autophagy in specific organs or tissues, is urgently needed.

One of the most promising autophagy inducers currently used preclinically is the Tat-Beclin peptide, discovered in the Beth Levine laboratory [19]. Tat-Beclin (T-B) is a 24 amino acid peptide comprised of a Tat cell penetrating sequence and a sequence derived from BECN1 (Beclin 1). BECN1 is a protein involved in autophagy as a component of the class 3 PI3K complexes 1 and 2 [20]. The molecular target of the T-B peptide is GAPR-1/GLIPR2 (Golgi-associated plant pathogenesis-related protein 1). The Tat-Beclin peptide is proposed to promote the release of BECN1 from the Golgi, where it is inactive in autophagy, to non-Golgi subcellular compartments, resulting in enhanced early autophagosome formation, and it may promote autophagy via other unknown mechanisms [19]. A recent study using model membranes and measuring membrane docking and enzyme activity showed that T-B can directly facilitate membrane docking and activate the PI3K complexes 1 and 2 [21]. The T-B peptide has been shown to induce autophagy both in vitro and in vivo and its therapeutic potential has been proved in various preclinical models of disease, such as viral infections [19,22], neuro-aging [23], sepsis [24] and cardiac hypertrophy [25], to name few examples. Yet, in most of these studies, the peptide must be injected daily to achieve therapeutic effects, due to its fast degradation in vivo. On the other hand, overinduction of autophagy by the T-B has been linked to an Na^+^/K^+^-ATPase-dependent cell death, termed autosis [26].

Different strategies have been evaluated to increase the stability and half-life of therapeutic agents, including peptide and protein therapeutics [27]. Modification of the peptide/protein structure [28], bioconjugation of peptides/proteins with polyethylene glycol (PEG) or PEG alternatives [29] and incorporation of the peptide/protein in drug delivery systems have been described [30,31,32,33]. Nanoparticles (NPs) can be used as drug delivery systems (DDS), which are used to carry therapeutic agents in vivo to increase efficacy and safety. This is achieved by: (a) improving the pharmacokinetic properties of drugs with a narrow therapeutic index, (b) enhancing intracellular delivery of active agents, which diminishes the need for a higher dose to achieve equal therapeutic effects and (c) passive or active targeting of the tissues/organs/cells that are in most need, resulting in higher therapeutic efficacy while sparing other tissues. The benefits of using nanoparticles for delivery of therapeutic agents in the liver has been proven in numerous studies reviewed elsewhere [34,35,36]. As an example, treatment with mPEG-PLGA polymeric particles loaded with the autophagy inducer rapamycin markedly improved hepatic steatosis and liver injury in a high fat diet mouse model of NAFLD compared to the free drug [37]. Of note, rapamycin is a poorly soluble drug with limited bioavailability, and its clinical use is limited by side effects [38]; it can therefore benefit from incorporation in a drug delivery system for improved safety and efficacy.

In this preliminary study, we hypothesized that combining the autophagy-inducing peptide with polymeric particles used as a biodegradable drug delivery system would protect it from degradation, facilitate intracellular peptide delivery and facilitate drug targeting in vivo. In addition, we examined the therapeutic potential of this system in an in vitro model of NAFLD and its biodistribution in healthy and obese mice. Collectively, this study paves the way for the development of novel tools to modulate autophagy in specific organs, which will ultimately contribute to the translation of autophagy-modulating therapeutics from the bench to the bedside.

## 2. Materials and Methods

### 2.1. Preparation of Tat-Beclin Polymeric Particles

Tat-Beclin polymeric particles were produced following a two-step process. First, racemic Poly(L-Lactide)/Poly(D-Lactide) (MW = 45–80 kDa, Merck, Zug, Switzerland) particles (NPs) were synthetized by a surfactant-free nanoprecipitation method adapted from Fessi et al. [39]. Briefly, the polymer was dissolved at 2% (*w/v*) in acetone (Carlo Erba, Val -de-Reuil, France). For preparation of near infra-red fluorescent NPs, XenoLight DiR (Perkin Elmer, Waltham, MA, USA, 125964) was added in the organic phase. The organic phase was then poured drop by drop into an aqueous phase composed of water and ethanol (Carlo Erba, Val-de-Reuil, France) under light stirring. Both organic solvents were then evaporated under low pressure in a rotary evaporator (Rotavapor R210, Buchi, Flawil, Switzerland) at 30 °C. Final NP suspension was diluted, if needed, with water, to a final concentration of 3% *w/v* and stored at 4 °C. One hundred and fifty microliters of the NP suspension was weighed in triplicates and dried overnight at 55 °C, to determine the proportion of PLA polymer into the suspension (*w/v*) (solid content) as an indirect measure of the yield of synthesis.

Then, peptides were adsorbed on particles’ surface. The peptides used were the random coils [19] Tat-Beclin L11 (T-B) (Novus Biologicals, Centennial, CO, USA, NBP2-49886) or the Tat-Scrambled L11s (T-S) (Novus Biologicals, Centennial, CO, USA, NBP2-49887), diluted in a tris(hydroxymethyl)aminomethane (TRIS) buffer. An equal volume of peptide solution was mixed with a suspension of NPs of 0.5% (*w/v*). The suspensions were mixed for 2 h at room temperature under 360° rotation. To reach a 300 mOsmol/L osmolarity for intravenous injections in mice, a 6% (*w/v*) solution of mannitol (Sigma Aldrich, Saint-Quentin-Fallavier, France, 69-65-8) was used to dilute the nanoparticles in the desired final solid content of 0.25% *w/v*.

### 2.2. Physicochemical Characterization of Tat-Beclin NPs

For colloidal characterization of NPs, 15 microliters was dissolved in a 0.22 µm filtered 1 mM NaCl solution. The NP hydrodynamic diameter (Z-average) and size distribution defined by the polydispersity index (PDI) were evaluated by dynamic light scattering (DLS) using a Zetasizer Nano ZS Plus (Malvern Panalytical, Malvern, UK). Zeta potential was calculated by measuring the NP electrophoretic mobility using the same apparatus, a Zetasizer Nano ZS.

To determine the amount of non-adsorbed T-B or T-S, 130 microliters of NP suspension was centrifuged for 10 min at 5000× *g* to pellet the NPs. The supernatant was collected and centrifuged for another 5 min at 16,000× *g* to pellet any remaining particles. The supernatant was transferred into a 96-well plate in duplicates for non-adsorbed peptide quantification using the Pierce Coomassie Plus (Bradford) Protein Assay reagent (ThermoFisher Scientific, Illkirch-Graffenstaden, France, 1856209), using bovine serum albumin (BSA) or T-B for the calibration curve. The adsorption efficiency and peptide loading on NPs were calculated according to the formulas:

$$Adsorption\;efficiency\;\left(\%\right)=100\times \frac{\left(Starting\;peptide\;concentration-non\;adsorbed\;peptide\;concentration\right)}{Starting\;peptide\;concentration}$$

$$Peptide\;loading\;\left(\%\right)=100\times \frac{Adsorbed\;peptide\;mass}{\begin{array}{c} Total\;PLA\;NP\;mass \end{array}}$$

Morphological characterization of NPs was performed with scanning electron microscopy (SEM). Briefly, a drop of NPs was diluted in water to a final concentration of 0.05% (*w/v*) solid content. The suspension was applied on glass coverslips and stuck on metal stubs before air drying under reduced pressure. The dried samples were then sputtered by 10 nm of copper with a metallizer (BAL-TEC MED020, Leica Microsystems, Nanterre, France) before SEM observations (MERLIN VP Compact, Zeiss, CTµ, Centre Technologique des Microstructures, Lyon 1, France) with an acceleration voltage of 10 kV.

### 2.3. Cell Culture and Treatments

Human epithelial adenocarcinoma cells (HeLa) and Human hepatocellular carcinoma/hepatoblastoma (HepG2) cells were acquired from Cellulonet (Lyon, France) and grown in Dulbecco’s modified Eagle medium Glutamax (DMEM Gibco, ThermoFisher Scientific, Illkirch-Graffenstaden, France) and supplemented with 10% fetal bovine serum (FBS Gibco, ThermoFischer Scientific, Illkirch-Graffenstaden, France) without antibiotics in a humidified atmosphere of 95% air/5% CO_2_ at 37 °C.

For evaluation of autophagy modulation by Western blot, cells seeded into 6-well plates were treated with T-B and T-S at a final concentration of 20 µM for up to 6 h or at 2 µM for 24 h. NP T-B and NP T-S were used at a final concentration of 2 µM for 16 up to 72 h. For autophagic flux experiments, bafilomycin A (ApexBio, Huston, TX, USA, 88899-55-2) was added at a final concentration of 50 nM in the last 2 h of incubation.

### 2.4. Evaluation of Autophagy and Autophagic Flux In Vitro

Modulation of autophagy was evaluated by protein expression of two common autophagy markers: MAP1LC3/LC3, which associates with the autophagosomes when in the lipidated form (LC3-II), and SQSTM1/p62 (sequestosome 1), which is an autophagic cargo protein. LC3 is the most widely monitored autophagy-related protein. It is initially synthesized as the unprocessed form, proLC3, which is converted into its proteolytically processed, cytosolic form LC3-I, and when autophagy is induced, it is finally conjugated to phosphatidylethanolamine (PE) to form LC3-II. LC3-II is the only protein marker that is reliably associated with completed autophagosomes [3]. SQSTM1/p62 is a protein that serves as a link between LC3 and ubiquitinated substrates. p62 and p62-bound polyubiquitinated proteins become incorporated into the completed autophagosome and are degraded in autolysosomes, thus serving as a marker of autophagic degradation [3]. Therefore, the conversion of LC3-I to its autophagosome-associated form LC3-II, as well as the degradation of p62, both normalized to a housekeeping protein such as ACTB (actin), can be assessed as a means of monitoring autophagy [40]. For example, the quantification of LC3II/ACTB and p62/ACTB can be used to monitor autophagy [41].

An increase in LC3-II/ACTB may be interpreted as either an increased formation of autophagosomes or a decreased degradation of autophagosomes. To evaluate whether an experimental treatment is inducing autophagy rather than blocking autophagosome degradation, autophagic flux experiments using bafilomycin A_1_ (Baf A) need to be performed. Baf A is a specific inhibitor of the vacuolar type H^+^-ATPase (V-ATPase) and inhibits the acidification of organelles containing this enzyme, such as lysosomes and endosomes, and thus indirectly prevents autophagosome clearance [42]. Therefore, in Baf-A-treated cells, the LC3-II levels and the LC3-II/ACTB ratio would increase compared to untreated cells. If an experimental treatment induces autophagy, then: (a) LC3-II levels will increase and (b) the increase should be even greater when bafilomycin is used concomitantly [41].

### 2.5. Immunoblotting and Antibodies

To prepare protein extract for immunoblot analysis, cells were washed twice with cold phosphate-buffered saline (PBS), scraped from the dishes and lysed at indicated time points in Radioimmunoprecipitation Assay (RIPA) buffer (ThermoFisher Scientific, Illkirch-Graffenstaden, France, 89901) supplemented with inhibitors of phosphatases, proteases and ethylenediaminetetraacetic acid (EDTA) (ThermoFisher Scientific, Illkirch-Graffenstaden, France, 78440). Lysates were centrifuged at 15,000× *g* for 20 min at 4 °C. Protein concentration of the supernatants was measured using the Pierce BCA Protein Assay Kit (ThermoFischer Scientific, Illkirch-Graffenstaden, France, 23227). Samples were subsequently diluted to an equal total protein concentration and supplemented with Laemmli Sample buffer (Biorad, Hercules, CA, USA, 1610747) and 2-mercaptoethanol (BioRad, Hercules, California, USA, 1610710). Samples were stored at −20 °C or run immediately on gels. Protein samples were run on SDS-PAGE precast gels (4–15%) (BioRad, Hercules, CA, USA, 4561086). Gels were blotted onto nitrocellulose membranes (BioRad, Hercules, CA, USA, 1704159) by transfer using the BioRad Trans-Blot Turbo Transfer System. The membranes were then blocked by nonfat dry milk solution (5%) in PBS with Tween 20 (0.1%) and incubated in desired primary antibody overnight at 4 °C. Primary antibodies that were used were anti-LC3B (Sigma Aldrich, Saint-Quentin-Fallavier, France, L7543), anti-actin (Sigma Aldrich, Saint-Quentin-Fallavier, France, A2066) and anti-SQSTM1/p62 (Abcam, Cambridge, UK, ab56416). Membranes were then washed with PBS-Tween 20 (0.1%) before incubating with peroxidase-conjugated secondary antibodies (Jackson ImmunoResearch, Cambridgeshire, UK, 115-035-062 and 211-035-109) for 1 h at room temperature. Blots were revealed with Western ECL substrate (BioRad, Hercules, CA, USA, 1705060). All band intensities were quantified using ImageJ 1.46r (National Institutes of Health, Bethesda, ML, USA) and the ratio of LC3B-II to actin was calculated as a readout for autophagy.

### 2.6. Cell Viability and Cytotoxicity Evaluation

For cell viability experiments using the Presto assay, HeLa and HepG2 cells were seeded in a 96-well transparent bottom-black plate. The next day medium was renewed, and cells were treated with a few microliters of T-B, T-S or NP, NP T-B and NP T-S for another 24 h, at a 2 µM final concentration. At the end of the incubation period, Presto blue (Invitrogen, ThermoFisher Scientific, Illkirch-Graffenstaden, France, A13261) was added for another 15 min, while cells were kept in the incubator. Fluorescence intensity was measured by excitation at 560 nm and emission at 590 nm using a microplate reader (Infinite M1000, Tecan, Männedorf, Switzerland). Blank (no cell) control values were subtracted from all measurements and cell viability was evaluated as a percentage related to the untreated control (positive control). For lactate dehydrogenase activity (LDH) assay (Cytotoxicity Detection kit Plus, Roche, Basel, Switzerland, 04744926001), cells were seeded in transparent 96-well plates and treated as in the Presto assay. After 24 h, control cells were treated with either lysis buffer or medium only, which represent positive (lysed cells) and negative (healthy cells) controls, respectively. Cells were subsequently treated with 100 μL/well of Reaction Mixture reagent for 10 min at RT. Then, 50 μL/well of Stop solution was added prior to absorbance measurement at 490 nm using the Tecan microplate reader. Blank (no cell) control values were subtracted from all measurements and cell cytotoxicity was evaluated as a percentage related to the lysed positive control group.

### 2.7. Induction of Steatosis in Cells and Quantification of Intracellular Lipid Droplets

HeLa cells were seeded at 15,000–20,000 cells/well on chambered slides (Ibidi, Gräfelfing, Germany, 81201). To establish the in vitro cell model of steatosis, cells were incubated with a combination of oleic (Sigma Aldrich, Saint-Quentin-Fallavier, France, O1008) and palmitic acid (Sigma Aldrich, Saint-Quentin-Fallavier, France, P0500) (OA/PA 0.8 mM/0.4 mM) for 24 h. Fatty acids were withdrawn, the medium renewed and the cells were then treated with T-B or NP T-B and NP for another 16 h. T-B was used at 10 µM and NP T-B at a concentration of 1 µM. Cells were then washed and fixed with 4% paraformaldehyde (*w/v*) in PBS for 15–20 min at room temperature. Cells were then stained with BODIPY (493/503) (Invitrogen, Fisher Scientific, Illkirch-Graffenstaden, France, D3922) (4 µg/mL) and DAPI (Sigma Aldrich, Saint-Quentin-Fallavier, France, D9542) (2 µg/mL) for 20–30 min. Slides were mounted using a few drops of Vectashield mounting medium (Vector Laboratories, Newark, CA, USA, H-1400) on coverslips. Samples were allowed to cure overnight in the dark at 4 °C and then imaged in a confocal microscope, Nikon LSM710 (PLATIM, Lyon, France). Number of lipid droplets was quantified with ImageJ 1.46r (National Institutes of Health, Bethesda, ML, USA) and Icy software (Institute Pasteur and France Bioimaging group, France). LDs were counted in at least 250–300 cells per condition. In ImageJ, a Triangle threshold was applied, and LDs were quantified using the Analyzing particles tool (size (μm^2^): 0.2 to infinity and circularity 0.2–1). In ICY, Scale 2 and sensitivity 20 were selected as the best settings in the Spot detection tool.

### 2.8. Preparation of Cell Samples for Conventional Electron Microscopy

HeLa cells were seeded in 12-well plates and cultured overnight. The next day cell medium was renewed, and cells were treated with NP or NP T-B for a total of 43 h, with/without bafilomycin A (Baf A) 50 nM added at the final 2–3 h of co-incubation. At the end of the incubation period, culture medium was removed, and cells were fixed with 2% glutaraldehyde in 0.2 M (4-(2-hydroxyethyl)-1-piperazineethanesulfonic acid) (HEPES) buffer, pH 7.4 for 20 min at room temperature. The cells were then scraped gently and transferred to Eppendorf tubes and pelleted at 16,000× *g* for 5–10 min at 4 °C. Cell pellets were left in fixative for a total of 2 h at 4 °C. Subsequently, fixative was removed, and tubes were filled immediately with 0.2 M HEPES buffer, pH 7.4, and stored at 4 °C until further processing. Pellets were post-fixed in OsO_4_ before being dehydrated in increasing concentration of ethanol and finally in acetone. Pellets were then embedded in epon. Sections were cut with a diamond knife on an ultramicrotome at 70 nm thickness and sections were picked up on the EM grids and stained with uranyl acetate and lead citrate. Grids were imaged in a JEM-1400 Plus Transmission Electron Microscope (Jeol, Tokyo, Japan).

### 2.9. Identification of Organelles in Transmission Electron Microscope (TEM) Images

To identify the type of organelles that enclosed NPs, early and late autophagic structures, endosomes, lysosomes and other vesicles were defined following these guidelines [43,44,45,46,47]:

- Autophagosomes have a diameter between 500 nm and 1.5 mm, have a double limiting membrane and enclose cytoplasmic cargo (for example, glycogen, ribosomes, endoplasmic reticulum or mitochondria). The double membrane, however, may not always be visible in EM sections;
- Late autophagic structures, for example autolysosomes, have a single limiting membrane but still contain cytoplasmic cargo. Their cargo appears darker than in autophagosomes, and has partially disintegrated morphology;
- Lysosomes have median diameters of 500–800 nm, often have a characteristic fingerprint or onion-like internal membrane structures, are filled with tiny granular matrix and appear dark in TEM images;
- Endosomes have a single limiting membrane and contain endocytosed material (for example nanoparticles), varying numbers of 50–100 nm internal vesicles, but no cytoplasmic cargo;
- Amphisomes (endosomes fused with autophagosomes) have a single limiting membrane and enclose both endocytosed material, 50–100 nm internal vesicles and cytoplasmic cargo.

### 2.10. Animal Groups and Handling

The experimental procedures and protocols of this study were approved by the ethics committee of the French ministry (APAFIS#27694-20200823221679 v8) and were in accordance with the European guidelines for the use of laboratory animals for scientific purposes. Five-week-old Crl:SKH1-Hrhr (SKH1) and B6.Cg-Lep^ob^/J (ob/ob) male mice were bought from Charles Rivers (L’Arbresle, France) and housed at the pathogen-free animal facility AniCan of the Centre Leon Berard (Lyon, France) (agreement n° D693880202). Mice were housed under specific pathogen-free (SPF) conditions (12 h light/12 h dark cycle at 22 ± 2 °C and 60 ± 5% relative humidity) and had ad libitum access to standard chow and water.

### 2.11. In Vivo Imaging by Tomography

After a habituation period of 2 weeks, mice were split in groups and anaesthetized one by one with isoflurane. Mice were immediately injected in the retro-orbital vein with 120 µL of either free fluorophore (Xenolight DiR-Fluorescent Dye, PerkinElmer, Waltham, MA, USA, 125964), fluorescent nanoparticles (NP DiR, Near Infra-Red Fluorescent i-Particles^®^, Adjuvatis, France) or fluorescent Tat-Beclin nanoparticles (NP DiR T-B) of 0.25% *w/v* solid content. Whole-body fluorescence was recorded immediately after injection and at 2 or 3 h, 24 h, 48 h, 72 h and 7 days after injection using the FMT4000 fluorescence tomography imaging system (Perkin Elmer, Waltham, MA, USA) and while mice were kept under anesthesia. The tomographer was calibrated with a known concentration of fluorescent NP, allowing a direct quantification of fluorophore in the selected regions of interest. The percentage values reported were calculated from the 5 min timepoint, when the maximal fluorescence was observed.

For the ex vivo organ biodistribution studies, SKH1 or ob/ob mice were injected with soluble fluorophore (DiR), NP DiR or NP DiR T-B, sacrificed after 24 h, and major organs (liver, spleen, pancreas, kidneys) were collected and imaged in the FMT4000.

## 3. Results

### 3.1. Design and Characterization of Tat-Beclin NPs

**Figure 1:**
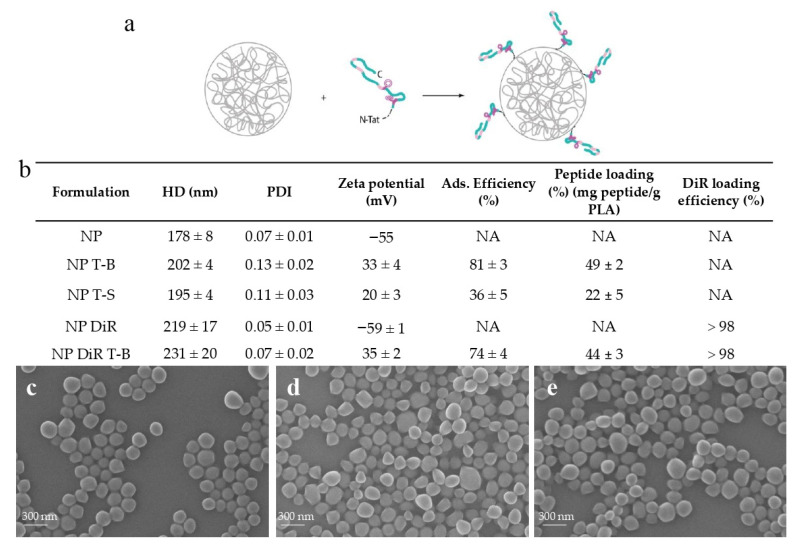
Physicochemical characteristics and morphology of peptide formulations based on PLA particles. (**a**) The Tat-Beclin (T-B) peptide or Tat-Scrambled (T-S) control peptide was adsorbed on biodegradable particles (NP) or fluorescent particles (NP DiR), made of poly(L-Lactide)/Poly(D-Lactide). (**b**) Hydrodynamic diameter (HD) (Z-average), polydispersity index (PDI), zeta potential, adsorption efficiency, peptide loading and DiR encapsulation efficiency of the prepared formulations. Values are means ± SD of three measurements pooled from at least three independent experiments. SEM micrographs of plain particles (NP) (**c**), Tat-Beclin particles (NP T-B) (**d**) and Tat-Scrambled peptide control particles (NP T-S) (**e**). Scale bar: 300 nm.

Considering the context- and tissue-dependent role of autophagy in health and disease, targeted autophagy induction in specific organs or tissues, while sparing others, could be of clinical interest. In this preliminary study, we decided to formulate the Tat-Beclin peptide in biodegradable particles made of amorphous poly (Poly(L-Lactide)/Poly(D-Lactide)) [47], a Food and Drug Administration (FDA)-approved polymer, for targeted induction of autophagy in vivo. Polymeric particles (NPs) were prepared by nanoprecipitation without the use of surfactants, and the Tat-Beclin (T-B) or Tat-Scrambled (T-S) control peptide was subsequently adsorbed on NPs. Adsorption of the T-B and the T-S peptide resulted in a slight increase in particle size (from 178 ± 8.0 nm for NPs to 202 ± 4.0 and 195 ± 4.0 nm, respectively) (Figure 1b). NPs, NPs with T-B (NP T-B) and NPs with T-S (NP T-S) had homogeneous size, as confirmed by SEM observations (Figure 1c–e). In addition, the particles became positively charged with the addition of the T-B or T-S peptide, as assessed by zeta potential values of +33.0 ± 4.0 mV and +20.0 ± 3.0 mV, respectively. Adsorption efficiency was 81.0 ± 3.0% for the NP T-B and less optimal for the NP T-S (36.0 ± 5.0%), while peptide loading was 49 ± 2.0% and 22 ± 5.0%, respectively (Figure 1b). With further optimization of this NP T-S formulation, a higher peptide loading could be achieved but the amount of unadsorbed T-S also increased (data not shown). We therefore decided to keep this less optimal NP T-S formulation considering that: (a) Tat-Scrambled should not have an effect on autophagy and (b) NP T-S are a useful control, as they have similar physicochemical properties as NP T-B.

For in vivo experiments, fluorescent DiR particles were prepared, and the T-B was subsequently adsorbed on their surface. Encapsulation of the fluorophore resulted in an increase in the size of particles (219 ± 17.0 nm), which was further increased by the adsorption of T-B, resulting in particles with a mean size of 231 ± 20.0 nm. Surface charge and adsorption efficiency of NP DiR T-B were similar to NP T-B (Figure 1b).

Overall, we successfully developed and characterized reproducible Tat-Beclin- and Tat-Scrambled-based formulations. No change in size, PDI and zeta potential was observed when the formulations were stored at 4 °C for up to 3 days (data not shown). For unmodified particles, stability was almost one year (data not shown). For subsequent experiments, the formulations were freshly prepared or prepared one day in advance before being used in vitro or in vivo.

### 3.2. Evaluation of Autophagy in HeLa Cells

**Figure 2:**
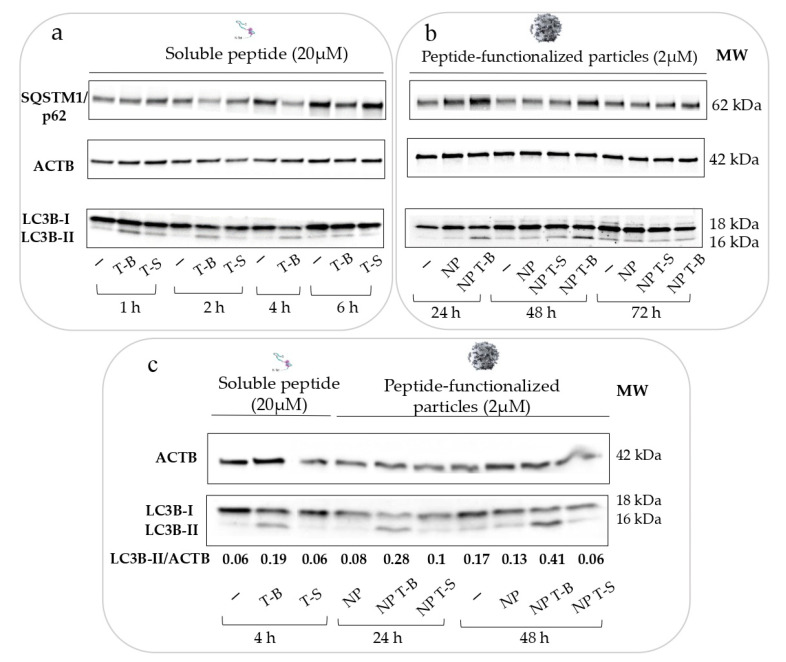
Particles formulated with the Tat-Beclin peptide modulate autophagy (taking LC3-II as a marker for autophagy) in a sustained and delayed manner, and at a ten times lower concentration compared to the soluble peptide in HeLa cells. (**a**) Autophagy modulation by the soluble Tat-Beclin peptide in HeLa cells over 6 h. HeLa cells were treated with 20 µM of soluble peptide for the respective time points before cell lysis, protein extraction and Western blot analysis of p62 and LC3B as autophagy markers. (**b**) Autophagy modulation by the functionalized particles (NP T-B or NP T-S at 2 µM) or plain particles (NP) at 24, 48 and 72 h post treatment. (**c**) Autophagy modulation by the soluble T-B or NP T-B at their peak of autophagic activity (4 h for the soluble peptide, 24 and 48 h for the NP T-B) and quantification of autophagy modulation (LC3B-II/ACTB ratio). All blots are representative of at least three independent experiments.

To verify that the Tat-Beclin peptide is inducing autophagy, HeLa cells were incubated with 20 µM of Tat-Beclin (T-B) or Tat-Scrambled (T-S) peptide for up to 6 h (Figure 2a), according to the manufacturer’s instructions. Modulation of autophagy was followed by immunoblot of two common autophagy markers: MAP1LC3/LC3, which associates with the autophagosomes when in the lipidated form (LC3-II), and SQSTM1/p62 (sequestosome 1), which is an autophagic cargo protein. An increase in LC3-II band intensity was observed at 1, 2 and 4 h post treatment (Figure 2a), suggesting induction of autophagy by the T-B peptide; the effect waned at 6 h post treatment. p62 tended to decrease at 2, 4 and 6 h, which may suggest activation of autophagic flux [3]. Apart from a small increase in LC3-II at 1 h post treatment, the Tat-Scrambled peptide did not alter LC3-II levels, and it did not affect p62 levels. Overall, T-B was shown to modulate LC3-II and p62 levels, and this effect was short-lived (lasting from 1 to 4 h), because LC3-II and p62 levels were already similar to the untreated control at 6 h post treatment (Figure 2a).

We then evaluated the effect of NP, NP T-B or NP T-S on autophagy. Considering that NP internalization occurs approximately within the first few hours of incubation, we evaluated LC3 and p62 levels at 6, 16, 24, 48 and 72 h post treatment. The earlier timepoints (6 and 16) are not reported here, as they were single-time experiments performed to understand when the effect on autophagy starts post treatment with the particles. NP and NP T-S did not have an effect on autophagy, as observed by LC3-II levels, while an increase in LC3-II was seen already at 6 h and became more pronounced at 16, 24 and 48 h following treatments with NP T-B (Figure 2b). The effect on LC3-II started to normalize at 72 h. Thus, the formulation of the peptide on the surface of the particles does not compromise its ability to modulate autophagy. Intriguingly, no concomitant p62 decrease was observed with the NP T-B formulation; on the contrary, there was a p62 increase at 24 and 48 h compared to control.

We then set out to compare the autophagy modulatory effect of the T-B peptide and NP T-B at their peaks of activity: after 4 h of stimulation for the peptide and 24 or 48 h for the NP T-B. As seen in the blot (Figure 2c), treatment with T-B and NP T-B but not T-S, NP or NP T-S resulted in conversion of LC3-I to LC3-II. The ratio of LC3-II/ACTB was higher for NP T-B at 24 and 48 h (0.28 and 0.41, respectively) compared to T-B for 4 h (0.19), suggesting an enhanced effect on autophagy for the formulated peptide. It is worth noting that the concentration of the peptide formulated with particles was 2 µM, ten times lower compared to the concentration of the soluble peptide. Importantly, this ten times lower dose of the T-B peptide formulated with particles was associated with a greater enhancement of LC3-II accumulation compared to the soluble peptide.

### 3.3. Evaluation of Autophagic Flux in HeLa and HepG2 Cells

**Figure 3:**
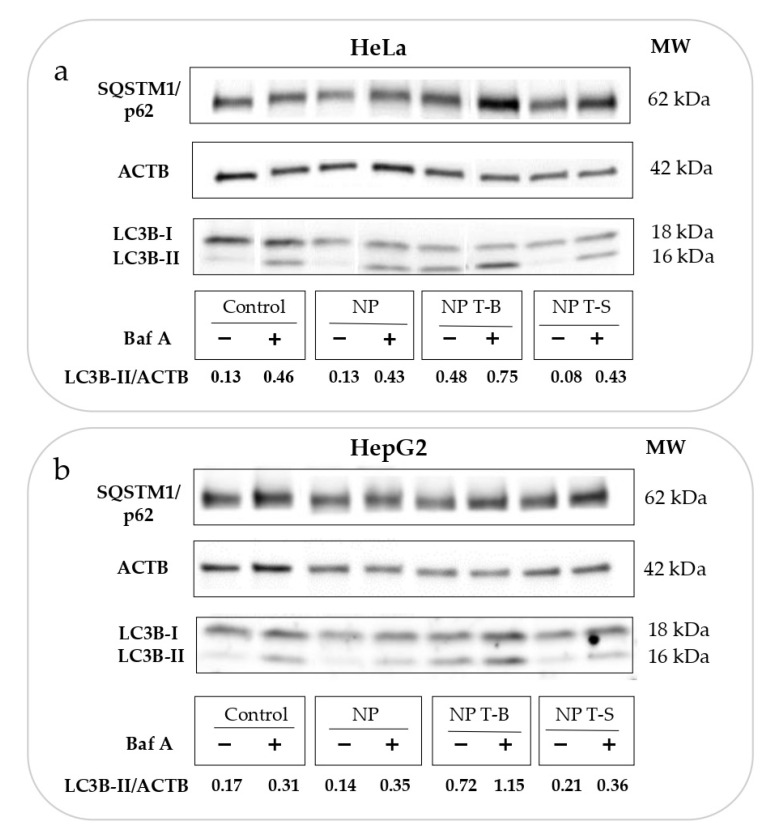
Evaluation of autophagic flux in (**a**) HeLa and (**b**) HepG2 cells treated with particle formulations. Cells were treated with NP T-B (2 µM) or NP T-S (2 µM) or NP for 24 h and bafilomycin A (50 nM) was added at the last 2 h of co-incubation. The LC3B-II/ACTB ratio was quantified in the blots. An increase in LC3B-II/ACTB in the presence of NP T-B and Baf A (NP T-B+) compared to Baf A treatment alone (Control+) denotes that NP T-B are original autophagy inducers. Both blots are representative of at least three independent experiments.

Autophagic flux experiments using bafilomycin are important to distinguish whether the enhanced LC3-II accumulation observed in the experiments reported above is a result of enhanced formation of autophagosomes (activation of autophagy and autophagic flux) or a blockage in autophagosome degradation. However, apart from its effect on autophagy, bafilomycin may interfere with the transport of endocytosed material to late endosomes [48]. Therefore, to avoid Baf A interfering with the intracellular trafficking of NPs, it was used only at the last 2 h of co-incubation of cells with NPs. Treatment with Baf A (Control+) enhanced the conversion of LC3-I to LC3-II and increased the ratio of LC3-II/ACTB (0.46) compared to untreated control HeLa (Control−) (0.13), proving sufficient block of autophagic flux (Figure 3a). NP T-B treatment (NP T-B−) increased the ratio LC3-II/ACTB (0.48), and this effect was further enhanced with simultaneous treatment with bafilomycin (NP T-B+) (0.75) (Figure 3a). Treatment with NPs alone (NP−) (0.13) and NP T-S alone (NP T-S−) (0.08) or in co-treatment with bafilomycin (NP+) (0.43) and NP T-S (NP T-S+) (0.43) did not alter the LC3-II/ACTB ratio compared to control alone (Control−) (0.13) or bafilomycin-treated control (Control+) (0.46), respectively. These results confirm that NP T-B are autophagy inducers, while unmodified or peptide control particles do not have an effect on autophagy.

Since autophagy is cell-type dependent, we proceeded to reproduce the autophagic flux experiments in a second cell line, the hepatoblastoma line HepG2, which is commonly used as a model for liver disease. Indeed, in this cell line, NP T-B but not NP or NP T-S increased the LC3-II/ACTB ratio (0.72 compared to 0.17 for control) (Figure 3b). It is also worth noting that while NP T-B treatment resulted in a significant increase in the LC3-II/ACTB ratio in HepG2 cells, the soluble T-B peptide used at the same concentration and for the same duration did not have such an effect. The scrambled peptide T-S, the T-S nanoparticles and unmodified NPs did not have an effect on autophagy (Supplementary Figure S1a,b). The LC3-II/ACTB ratio was significantly increased in NP-T-B-treated cells (NP T-B−) compared to untreated control cells (Control−) (Supplementary Figure S1c). Furthermore, there was a significant increase in the LC3-II/ACTB ratio of NP T-B plus Baf-A-treated cells (NP T-B+) compared to Baf-A-treated cells (Control+), proving autophagy induction by NP T-B (Supplementary Figure S1c). In sum, we showed that NP T-B but not NP T-S or NP can induce autophagy in two cell lines, HeLa and HepG2.

### 3.4. Evaluation of Viability in HeLa and HepG2 Cells

**Figure 4:**
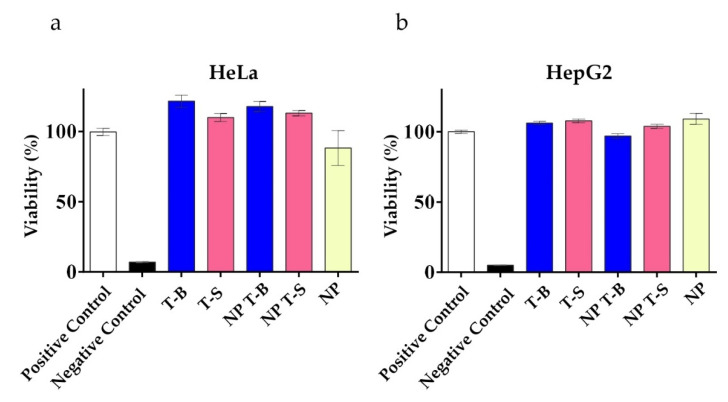
Viability of HeLa (**a**) and HepG2 (**b**) cells treated with Tat-Beclin particles (NP T-B), Tat-Scrambled particles (NP T-S), plain particles (NP) and T-B or T-S, as evaluated by a Presto assay. Cells were treated with the soluble peptides (T-B and T-S) and the different formulations (NP T-B and NP T-S) at a concentration corresponding to 2 µM of peptide for a total of 24 h. NP, NP T-B and NP T-S were used in the same particle concentration. Presto blue was added at the end of the incubation period for another 15 min and fluorescence intensity was measured by excitation at 560 nm and emission at 590 nm in a microplate reader. Viability percentages are reported to the untreated condition (positive control). Values are means ± s.e.m of three replicates out of three independent experiments.

Cellular viability was examined to evaluate whether the formulations were well tolerated. Viability, as an indirect measure of metabolic activity, was evaluated using a Presto assay in HeLa and HepG2 cells following a 24 h treatment with T-B, T-S or NP, NP T-B and NP T-S, similar to conditions used to evaluate autophagic activity. In HeLa cells (Figure 4a), treatment with T-B and T-S, either in soluble or particle form, was well tolerated and resulted in increased viability values compared to the positive control (untreated cells), probably due to the enhanced metabolic activity of cells treated with the different peptides or peptide formulations. In HepG2 cells (Figure 4b), metabolic activity was also slightly enhanced upon treatment with the different peptides or peptide formulations. An LDH cytotoxicity assay which is less dependent on metabolic activity was performed as a supplementary assay to the Presto assay (Supplementary Figure S2). Indeed, treatment with the peptides or the different formulations were not cytotoxic to HeLa (Supplementary Figure S2a) nor HepG2 cells (Supplementary Figure S2b).

### 3.5. Intracellular Fate of Autophagy-Inducing Particles

Transmission electron microscopy (TEM) can provide information on the uptake mechanisms and intracellular fate of nanoparticles [49]. For TEM imaging, HeLa cells were treated with NP or NP T-B for 43 h, at the same concentrations as in immunoblot experiments.

**Figure 5:**
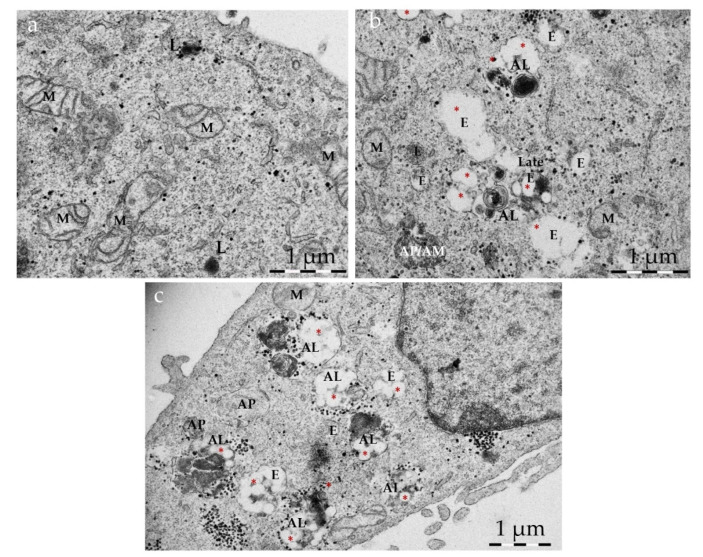
Intracellular accumulation of NP T-B and autophagic compartments. Representative TEM images of HeLa cells: untreated (**a**), treated with NP T-B (2 µM) (**b**) or NP T-B (2 µM) for a total of 43 h and bafilomycin A (50 nM) added at the last 2–3 h of co-incubation (**c**). At basal conditions (**a**), autophagic vacuoles were scarce but a few lysosomes (L) were present. Many endosomal compartments and some autophagosomes (AP) with a double membrane containing cytoplasmic cargo were observed in NP-T-B treated cells (**b**,**c**). Co-treatment with bafilomycin (**c**) resulted in a greater accumulation of autophagic compartments in NP T-B co-treated samples. NPs (denoted with red asterisks) were observed in endosomal (E), endolysosomal (EL) and autolysosomal (AL) compartments (**b**,**c**). Images are representative of at least 50 images for each treatment condition. AL: autolysosome; E: endosome; EL: endolysosome; L: lysosome; M: mitochondrion. Scale bar: 1 µm.

NPs were found inside vesicles, typically 1–5 NPs profiles in each (Supplementary Figure S3a), while for NP T-B there were typically 1–10 NP T-B profiles in each (Supplementary Figure S3b). Given the lack of cargo other than NPs inside these vesicles, they can be characterized as putative endosomes. For NP T-B, bigger endosomes were observed compared to NP-treated cells, enclosing more particles, either as distinct particles or in advanced stage of degradation, with no distinct limiting membrane between adjacent particles (Supplementary Figure S3b). There were more endosomes with particles in NP-T-B-treated cells compared to NP-treated cells, probably due to the cationic charge of NP T-B, which favors cellular uptake [50]. NP T-B were mostly found inside endosomes, endolysosomes and autolysosomes, but not early autophagosomes (Figure 5b,c). In control conditions, autophagic vacuoles were scarce (Figure 5a). In cells treated with NPs, autophagic vacuoles were also scarce, but increased with co-treatment with bafilomycin, as expected. In NP-T-B treated samples (Figure 5b), we could find more endosomal compartments compared to untreated cells and some autophagosomes, while in NP T-B co-treated with bafilomycin (Figure 5c), the frequency of late autophagic structures was further increased.

### 3.6. Evaluation of Intracellular Lipid Droplet Content in an NAFLD Cell Model

**Figure 6:**
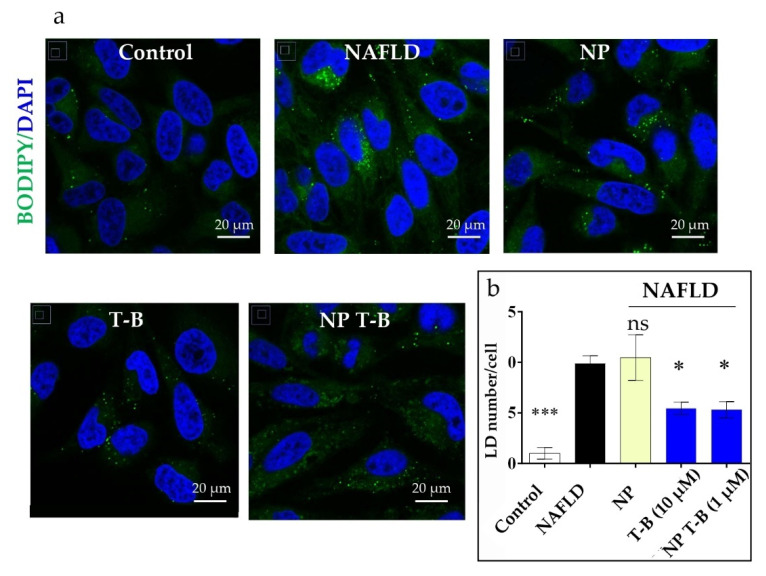
Effect of T-B and different formulation treatment on lipid droplet (LD) number in an NAFLD model in HeLa cells. (**a**) Representative images. HeLa cells were treated with fatty acids (OA/PA 0.8 mM/0.4 mM) for 24 h to induce LD formation and subsequently treated for 16 h with T-B (10 µM), NP T-B (1 µM) or NP. Fixed cells were stained with Bodipy (493/503) for lipid droplets (in green) and DAPI for nuclei (in blue) and imaged with a confocal microscope. Scale bar, 20 μm. (**b**) Quantification of lipid droplets (green dots) per cell for all conditions. LDs were quantified with the ICY software. Values are the means ± s.e.m. from replicates counting at least 250 cells. * *p* ≤ 0.05, *** *p* ≤ 0.001; ns: non-significant by one-way ANOVA and Dunnett’s multiple comparison test (all compared to NAFLD).

We hypothesized that if T-B and NP T-B can induce autophagy, they might also induce the selective degradation of lipids. In cells, lipids are stored within fat bodies, called lipid droplets (LDs), and their degradation is driven by lipolysis and autophagic processes (lipophagy) [7]. To induce lipid droplet formation in HeLa cells, we incubated cells with oleic and palmitic acid (2:1 molar ratio), as performed by Lim et al. [51], before treatment with the T-B peptide or NP T-B. The number of LDs was quantified in fluorescence microscopy images following staining of LDs with Bodipy (493/503). Quantification was performed using two types of imaging software, Image J and Icy, which both yielded similar results. HeLa cells have a low number of LDs at the basal level (control), and supplementation with fatty acids significantly increased the number of LDs (NAFLD) (Figure 6b). Overnight treatment with T-B at a concentration of 10 µM significantly decreased the number of LDs in the NAFLD model (Figure 6b). Treatment with NP T-B also resulted in a reduction in the LDs in the NAFLD cell model, at a ten times lower peptide concentration (Figure 6b). However, a different staining pattern was observed in the NP-T-B-treated cells compared to the T-B-treated ones. In NP-T-B-treated cells, big spherical droplets of high fluorescence intensity and a diffuse signal of lower intensity was observed in the cytoplasm, while in the latter, the diffuse cytoplasmic signal was absent (Figure 6a). To our knowledge, this is one of the few pieces of evidence of Tat-Beclin decreasing lipid droplet number in vitro.

### 3.7. In Vivo Biodistribution in SKH1 and ob/ob Mice

**Figure 7:**
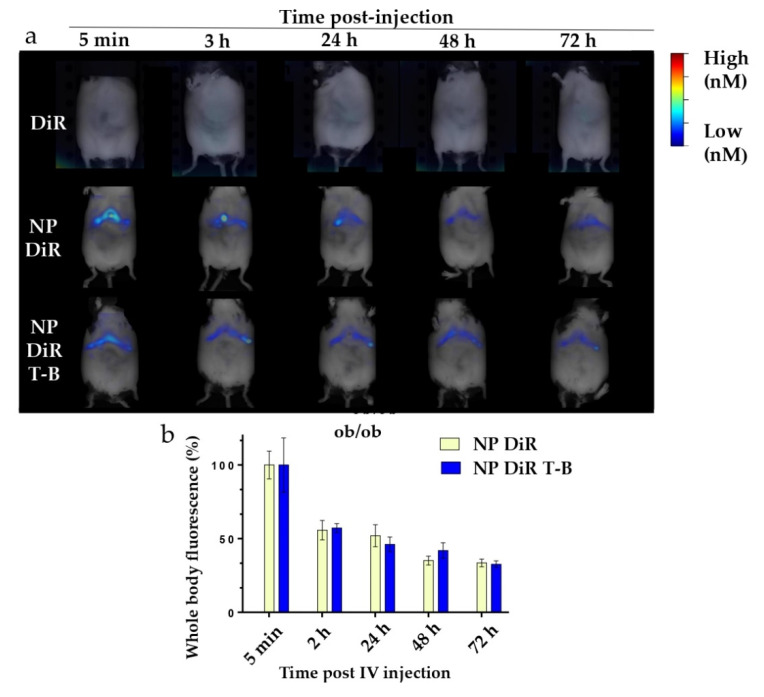

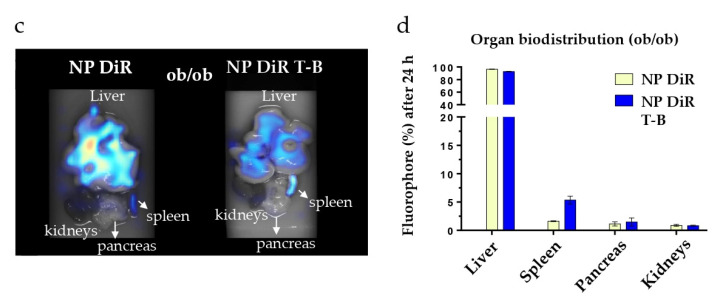
In vivo and organ DiR fluorescence imaging of genetically obese ob/ob mice following a single IV injection (120 µL) of soluble fluorophore or fluorescent particles (0.25% *w/v*). (**a**) Autophagy-inducing fluorescent particles (NP DiR T-B) and plain fluorescent particles (NP DiR) accumulate in the liver over the course of 72 h. Images are representative of scans from 3–4 mice per group. (**b**) Quantification of whole-body fluorescence up to 72 h post a single IV injection. The percentage values reported are calculated from the 5 min timepoint, when the maximal fluorescence was observed, and are reported as mean ± s.e.m. Results were analyzed using one-way ANOVA followed by Sidak’s multiple comparison test, which revealed no significant difference between the NP DiR and the NP DiR T-B group for all timepoints. (**c**,**d**) Organ biodistribution of fluorescent NPs 24 h after a single intravenous (IV) injection in ob/ob mice. *n* = 3–4 mice per group. Ex vivo DiR fluorescence imaging on main organs (liver, spleen, pancreas and kidneys) (**c**) and quantification of DiR fluorescence (**d**). The percentage values reported are calculated from total organ fluorescence and presented as mean ± s.e.m (**d**).

Targeting the liver with autophagy-inducing particles is highly desired in the context of NAFLD. Depending on their physicochemical properties, 30–99% of intravenously administered nanoparticles of size above 6 nm are sequestrated by the liver [52]. We thus injected our ~200 nm fluorescent NP DiR and NP DiR T-B in the retro-orbital vein of SKH1 and ob/ob mice. Biodistribution experiments were firstly performed in SKH1 mice which are hairless and therefore ideal for in vivo fluorescence tomography purposes. Biodistribution was then evaluated in a genetic model of obesity (ob/ob mice), characterized by obesity and a fatty liver already at 4 weeks of age. Whole-body fluorescence was recorded in anesthetized mice in the FMT4000 tomographer at various timepoints, from 5 min to 7 days (for SKH1 mice) or 3 days (for ob/ob mice) post a single injection with NP DiR or NP DiR T-B (Supplementary Figure S4a and Figure 7a, respectively). We could not obtain data on the ob/ob mice for 7 days after injection because the mice were already too obese to fit in the imaging cassette. Free fluorophore was observed in the liver of SKH1 mice 2 h after injection, but no signal was detected after 24 h, suggesting rapid clearance of the soluble fluorophore (Supplementary Figure S4a). The soluble DiR fluorophore was not detected in any of the ob/ob mice at any timepoints (Figure 7a). On the contrary, NP DiR and NP DiR T-B accumulated in the liver, and the signal was observed up to 72 h. Fluorescence gradually decreased over the course of several days, with approximately 50% of the initial signal persisting 24 h post injection (Supplementary Figure S4b). No significant differences in the biodistribution kinetics over the course of 7 days were observed between NP DiR and NP DiR T-B injected mice. Similar results were observed in ob/ob mice: approximately 50% of the initial fluorescence was still observed 24 h post injection and 30% after 72 h, for both NP- and NP-T-B-treated mice, and there were no significant differences between the two groups (Figure 7b). Ex vivo organ imaging 24 h after IV injection of fluorescent NP DiR and NP DiR T-B in SKH1 (Supplementary Figure S4c,d) and ob/ob mice (Figure 7c,d) revealed liver targeting with both formulations and increased spleen targeting of the NP DiR T-B compared to NP DiR in both mouse models.

## 4. Discussion

Induction of autophagy has gained increased attention as a therapeutic strategy for different diseases, including NAFLD. Evidence suggests that autophagy is downregulated in fatty liver [18] and that overnutrition interferes with autophagic and lipophagic functions [7]. In NAFLD patients, the number of autophagosomes and lipid-laden lysosomes (lipolysosomes) in liver increased with higher NAFLD activity score (NAS), and in liver sections of mice fed a high fat diet, there was a significant decrease in LD-containing macroautophagic intermediates, suggesting an impairment in lipophagy [15]. The liver can sequester systemically administered nanoparticles, rendering the latter an attractive tool to deliver therapeutic molecules in the context of liver pathologies. In this preliminary study our main goal was to develop and characterize autophagy-inducing particles for a liver-targeted induction of autophagy. We then evaluated their therapeutic potential in an in vitro NAFLD model and their biodistribution in healthy and obese mice after intravenous administration. To begin with, we successfully adsorbed the autophagy-inducing Tat-Beclin peptide onto biodegradable PLA particles in a reproducible manner. Surface charge of NPs before adsorption was highly negative, as assessed by its zeta potential value of −55 mV (Figure 1), due to the presence of the surface carboxylic end group of PLA, as these particles are produced without any surfactant or stabilizer. On the contrary, the T-B peptide sequence (Novus biologicals) contains six arginines, two lysines and two histidines, and is therefore characterized by a highly cationic isoelectric point (12.01 according to computational predictions using the Expasy ProtParam tool). In our conditions of adsorption in a low ionic strength buffer, T-B adsorption on the PLA NP surface may be mainly driven by electrostatic interactions, but can also be the result of hydrophobic and van der Waals interactions and hydrogen bonding, as previously described [53]. Different formulation conditions were tested to favor a high adsorption yield, while limiting particle aggregation. Previously, similar high-yield adsorption has been achieved with the same PLA particles and lysozyme [54] or poly-L-lysine [55] as cationic proteins. Resulting Tat-Beclin PLA particles (NP T-Bs) were cationic (+33 ± 4 mV), with a size slightly above 200 nm and a homogeneous size distribution. We then set out to evaluate the effect of soluble and particle-associated peptides over time on autophagy in vitro. In their study to characterize the Tat-Beclin peptide, Shoji-Kawata et al. treated HeLa cells for a similar length of time (3 h) as this study and highlighted autophagy induction by Western blot, evaluating the conversion of LC3-I to the lipidated form LC3-II [19]. Using the same method to evaluate autophagy, we found that the effect of T-B was strong yet short-lived and started to wane at 6 h post treatment. Our Western blot experiments in HeLa cells, using LC3 conversion as a readout for autophagy, revealed enhanced and sustained autophagy modulation (starting from 6 h up to 48 h) by NP T-B compared to the T-B peptide (up to 4 h), at a ten times lower dose. In a study by Zhang et al., PLGA-lecithin-PEG particles were loaded with a modified Tat-Beclin peptide, giving rise to Tat-vFLIP-α2 nanoparticles, and were tested in vitro for their ability to induce autophagy and kill HIV-infected cells. These particles had an average size of 147 nm, a +30 mV surface charge and a maximal loading capacity observed at ~15% (*w/w*). Human macrophages were treated with 10 µM of Tat-vFLIP-α2 nanoparticles for 8 h, then medium was renewed and autophagy monitored for an additional 48 h. It was found that treatment with Tat-vFLIP-α2 nanoparticles led to persistent autophagy induction, as evidenced by a significant increase in LC3-II/ACTB at 6, 12, 24 and 48 h and a significant decrease in p62/ACTB at 12, 24 and 48 h, both compared to the 0 h timepoint [22]. The LC3B/ACTB enhancement observed at 24 and 48 h in our study is in accordance with the study by Zhang et al., yet we used 2 µM of the formulated peptide compared to the 10 µM of formulated peptide used in the Zhang study. It is, however, worth noting that NP T-B increased the levels of another marker of autophagy, p62 in Hela cells, which is not in accordance with autophagy induction. While p62 levels decrease with autophagy induction using the Tat-Beclin peptide, p62 increases have been reported as a result of an enhanced autophagic activity. This could be due to a transcriptional upregulation resulting from enhanced autophagy or by activation of other cellular pathways [3]. Of note, p62 participates in proteasomal degradation, and its level may also increase when the proteasome is inhibited [3]. It would thus be interesting to evaluate mRNA levels of p62 in subsequent experiments, as well as monitor p62 mRNA and protein levels over longer timepoints.

The Tat peptide is thought to enter cells via endocytosis and membrane translocation mechanisms [56], yet when associated with nanoparticles, the endocytic uptake is the driving factor for entry [57]. However, bypassing the endocytic pathway and achieving direct cytoplasmic delivery has been described for liposomes of approximately 200 nm, on the surface of which a Tat peptide was covalently attached [58]. The discrepancies in these studies may be due to the different cell lines and nanoparticle types used. The mechanism of autophagy induction by T-B has been described by Shoji-Kawata et al. [18]. To be able to induce autophagy, the Tat-Beclin peptide needs to bind its target, GAPR-1, which is a Golgi-associated protein found in the cytoplasm, while it has also been proposed that T-B acts directly on the class 3 PI3K complex, thereby activating autophagy [21]. The enhanced effect of NP T-B on autophagy modulation compared to the soluble T-B peptide may be due to an enhanced intracellular delivery of NP T-B and/or protection of the T-B peptide from degradation in the NP-T-B-associated form. Intracellular delivery has been proven by EM observations of HeLa cells treated with NP T-B in this study. Additional mechanisms may also play a role, for example endosomal escape [56] of the T-B after NP T-B have been internalized in endosomes, but the exact mechanism needs to be proven in future experiments.

Nanomedicine has attracted increased attention since the 1960s, yet not so much is known on how nanoparticles interact with the autophagic machinery. Nanoparticles most often induce autophagy in vitro, and this induction of autophagy may be a protective mechanism by which cells tend to degrade what is foreign and aberrant [59]. Recently, interference with the autophagic and lysosomal pathways has been linked to nanomaterial toxicity [59]. For example, exposure to silver nanoparticles resulted in activation of autophagy, but the subsequent autophagosome–lysosome fusion was defective [60]. The authors proposed the interference of silver NPs with ubiquitination to have a role in defective autophagy and the resulting cytotoxicity [60]. In another study, non-biodegradable polymeric particles (with a hydrodynamic diameter of 54 nm and a charge of 41 mV) made of the FDA-approved Eudragit induced autophagy in a rat macrophage cell line. The particles also reached mitochondria and changed their morphology. The authors proposed the evaluation of cytotoxic effects and autophagy to be investigated for every type of carrier to be used for drug delivery [61]. In this study, the different formulations (NP T-B, NP T-S, NP) and the soluble peptides were all well tolerated, and no decrease in cell viability was observed in HeLa and HepG2 cells as evaluated by a Presto and a cytotoxicity assay. The enhanced viability observed with the Presto assay represents enhanced metabolic activity, which may be due to activation of autophagy and/or other signaling pathways related to nanoparticle uptake. The autophagic effects of NPs are dependent on their physicochemical properties (size, charge, dispersity), their concentration, and are also cell-type dependent [62]. In the present study at the concentrations tested, in two cell lines (HeLa and HepG2), LC3 accumulation associated with autophagy induction was observed after cell incubation with the NP T-B but neither with the formulated Tat-Scrambled peptide (NP T-S) nor plain particles (NP). We therefore conclude that the observed effect is due to the T-B, which is adsorbed on particles, and not because of some other property of the particles (size, charge or the presence of the Tat moiety). The role of NP T-B as inducer of autophagy was further confirmed in autophagic flux experiments, using bafilomycin A as an inhibitor of autophagosome degradation. Since T-S either in soluble or NP form did not induce autophagy, subsequent experiments were performed with NP T-B and unmodified NPs as control.

To elucidate the intracellular fate of NP T-B, we performed TEM. NP T-B were observed inside endosomal, endolysosomal and autolysosomal structures, but not autophagosomes. NP T-B were in various stages of degradation and often clustered together inside these structures. Compared to plain particles, more NP T-B were observed inside cells, which is expected due to the cationic charge of these particles, which interacts strongly with the negatively charged cell membrane [50]. As expected, endocytic compartments and autophagic vesicles were observed in the NP-T-B-treated samples, and co-treatment with bafilomycin further increased the presence of autophagic vesicles, as recently described in De Mazière et al. [63].

The therapeutic potential of the autophagy-inducing particles was subsequently investigated in an NAFLD model: HeLa cells supplemented with fatty acids to induce lipid droplet formation, as performed by Lim et al. [51]. The Tat-Beclin and Tat-Beclin particles decreased the number of lipid droplets in this model, suggesting that autophagy induction is a potent strategy to reduce intracellular lipid. Importantly, NP T-B decreased LDs at a ten times lower dose compared to the soluble peptide. The ability of T-B to induce LD breakdown has been described only once before in the literature [64], yet the effect of T-B may depend on the cell line and the lipid substrate used to mimic steatosis. Specifically, the Tat-Beclin peptide induced LD breakdown in RAW cells pulsed with acetylated low-density lipoprotein but not oleic acid as the lipid substrate [64]. In fact, the Tat-Beclin peptide surprisingly increased LD biogenesis when the lipid substrate was OA [64], underlying the complex relationship between autophagy induction, LD biogenesis and LD breakdown. Further experiments are needed to confirm whether the LD breakdown observed in our study is actually lipophagy. A limitation in this study was the use of HeLa cells as a model of NAFLD. We have repeated experiments using hepatoblastoma HepG2 cells, a cell line routinely used as a model for steatosis. However, in our hands this cell line was not well suitable for imaging experiments, as cells tended to form clusters. LDs were scarce in the middle of these cell clusters, while more abundant in the bordering cells. HeLa cells, on the contrary, formed a thin monolayer, and when induced with fatty acids, few, well-defined droplets were formed, which is ideal for imaging and LD quantification.

Induction of autophagy in the liver is desired in the context of NAFLD and NASH, to reduce intracellular lipid in hepatocytes [7] and to reduce inflammation in macrophages [65]. The liver of obese mice is distinctly different from the liver of healthy mice, which might influence nanoparticle biodistribution. A recent study highlighted that about 80% of genetically obese ob/ob fed with standard chow diet have steatosis and mild necroinflammation (presence of few lobular aggregates of inflammatory cells with or without apoptotic bodies) in the liver, but usually no fibrosis [66]. In addition, surface charge can influence particle biodistribution in vivo, owing to opsonization and altered uptake by phagocytic cells [67]. We thus set out to evaluate whether NP T-B can target the liver of normal and obese mice following a single intravenous injection and whether there is a difference in the biodistribution between NP-T-B- and NP-treated mice. When following NP T-B biodistribution over time, particle-associated fluorescence was observed in the liver three days after a single IV injection, in both SKH1 and obese mice. In SKH1 mice, no particle fluorescence was observed seven days following injection. Biodistribution of NP was similar to that of NP T-B in SKH1 and obese mice. The highly specific liver localization most likely results from the permeability of the capillaries in this organ, which exhibit no lamina propria and 100–200 nm channels along the endothelial wall [68]. A similar observation was described using poly(D,L-lactide-co-glycolic acid) (PLGA) as the polymer and adsorbed polyethyleneimine (PEI) as the cationic molecule on the surface to obtain positively charged PLGA nanoparticles. These 200 nm cationic particles accumulate in the liver and spleen after intravenous administration for at least 24 h [69]. The sustained accumulation of NP T-B in the liver is important for achieving therapeutic effects with a few doses, which is highly desired in a clinical setting. In the liver, autophagy induction has been shown to be beneficial for hepatocytes, Kupffer macrophages and liver sinusoidal endothelial cells (LSECs), while in stellate cells, it is believed to be fibrinogenic [70]. Given the low population of stellate cells in the liver [71], induction of autophagy in the whole liver is most likely beneficial. To elucidate in greater detail which organs are targeted by fluorescent NP and NP T-B, we isolated the major organs in the 24 h following a single IV injection. While NP accumulated mainly in the liver 24 h after IV injection, NP T-B accumulated mainly in the liver and spleen, in both models. The difference in the organ biodistribution is most likely due to the difference in the surface charge of NP and NP T-B. To further promote liver targeting and avoid spleen accumulation, galactosyl or folate or transferrin could be co-adsorbed on the NP T-B surface to facilitate active liver targeting. Co-adsorption of peptide or proteins has been reported before on such PLA particles, for example by p24 and RGD proteins on PLA NPs, for vaccine development purposes [72]. It is also worth noting that no toxicity (as evaluated by body weight monitoring and transaminase analysis) was observed in ob/ob mice injected twice per week with NP and NP T-B for a total of 5 weeks (Supplementary Figure S5), suggesting that repeated administration of the formulations is well tolerated. Finally, given the in vitro efficacy results and the proven liver targeting of NP T-B in obese mice presented in this study, the ability of NP T-B to activate autophagy and reduce lipid in the liver of obese mice requires further investigation.

## 5. Conclusions

Formulations of the autophagy-inducing Tat-Beclin peptide with polymeric PLA nanoparticles (NP T-B) were developed and characterized. In HeLa cells, NP T-B modulated autophagy at an enhanced and sustained manner and at a lower dose compared to the soluble Tat-Beclin peptide. Autophagic flux experiments in two cell lines confirmed that NP T-B induce autophagy. Electron microscopy imaging revealed that NP T-B accumulate in endosomal and autolysosomal compartments. In an in vitro model of NAFLD, NP T-B decreased the number of lipid droplets at a lower dose compared to T-B. The formulation of Tat-Beclin peptide onto biodegradable particles therefore allowed to reduce the effective peptide doses, for increased efficiency, probably by promoting cell uptake, while guaranteeing low toxicity. Liver targeting of NP T-B was observed in healthy (SKH1) and obese (ob/ob) mice, providing evidence that autophagy-inducing particles could be an exciting candidate as a therapeutic strategy for NAFLD and will require further investigation.

## Data Availability

Not applicable.

## Supplementary Materials

The following supporting information can be downloaded at: https://www.mdpi.com/article/10.3390/pharmaceutics14071379/s1, Figure S1: NP T-B induce autophagy in HepG2 cells; Figure S2: Tat-Beclin particles (NP T-B), Tat-Scrambled particles (NP T-S), plain particles (NP) and T-B or T-S did not negatively impact viability of HeLa (**a**) and HepG2 cells (**b**), as evaluated by an LDH cytotoxicity assay; Figure S3: Enhanced uptake of NP T-B particles compared to NP in HeLa cells; Figure S4: In vivo and organ DiR fluorescence imaging of SKH1 mice following a single IV injection (120 µL) of soluble fluorophore or fluorescent particles (0.25% *w/v*). Figure S5: Effect of repeated administration of NP and NP T-B on body weight (**a**), liver to body ratio (**b**) and liver transaminases of ob/ob mice.
