# Supplementary material for: Design and Evaluation of Autophagy-Inducing Particles for the Treatment of Abnormal Lipid Accumulation
Source: Pharmaceutics. 2022 Jun 29;14(7):1379. doi: 10.3390/pharmaceutics14071379 (PMC9318411; doi:10.3390/pharmaceutics14071379)
Supplement: Supplementary file 1 [file pharmaceutics-14-01379-s001.zip › pharmaceutics-1758387-supplementary.pdf]

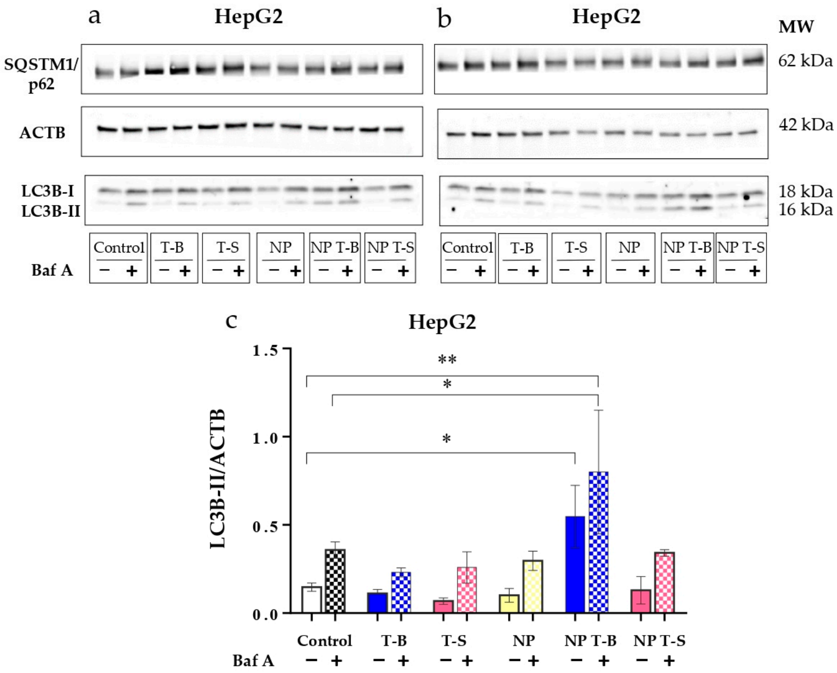

Figure S1. Comparison of autophagy induction by an equal dose of T-B and NP T-B in HepG2 cells, as evaluated by quantification of LC3B/ACTB. Representative blots (**a** and **b**) and quantification (**c**) of LC3B-II/ACTB of two independent experiments in HepG2 cells. HepG2 cells were treated with T-B, T-S (in soluble or NP associated form, 2 μM) or plain particles for 24 h and Baf A (50 nM) was added at the last 2 h of incubation. (**c**) Quantitation of western blot analysis of LC3-II/ACTB ratios. Bars represent mean ± s.e.m and results were analyzed using one-way ANOVA followed by an uncorrected Fisher's LSD multiple comparison test (* $p \leq 0.05$, ** $p \leq 0.01$).

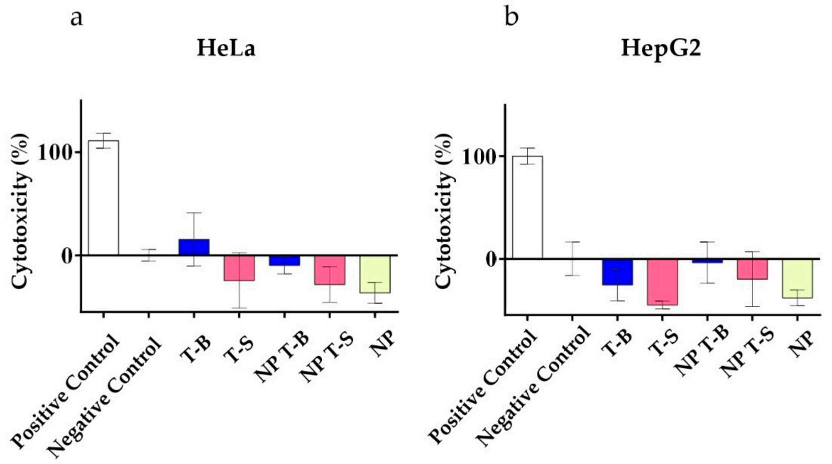

Figure S2. Cytotoxicity of HeLa (**a**) and HepG2 (**b**) treated with Tat-Beclin particles (NP T-B), Tat-Scrambled particles (NP T-S), plain particles (NP) and T-B or T-S, as evaluated by an LDH cytotoxicity assay. Cells were treated with the soluble peptides (T-B and T-S) and the different formulations (NP T-B and NP T-S) at a concentration corresponding to 2 μM of peptide for a total of 24 h. NP, NP T-B and NP T-S were used in the same particle concentration. Positive control cells were treated with lysis buffer and Negative control cells with cell medium. Cells were subsequently treated with Reaction Mixture reagent for 10 min at room temperature and Stop solution was added prior to absorbance measurement at 490 nm using the Tecan microplate reader. Cytotoxicity percentages are reported to the untreated condition (negative control). Values are means ± s.e.m of three replicates out of three independent experiments.

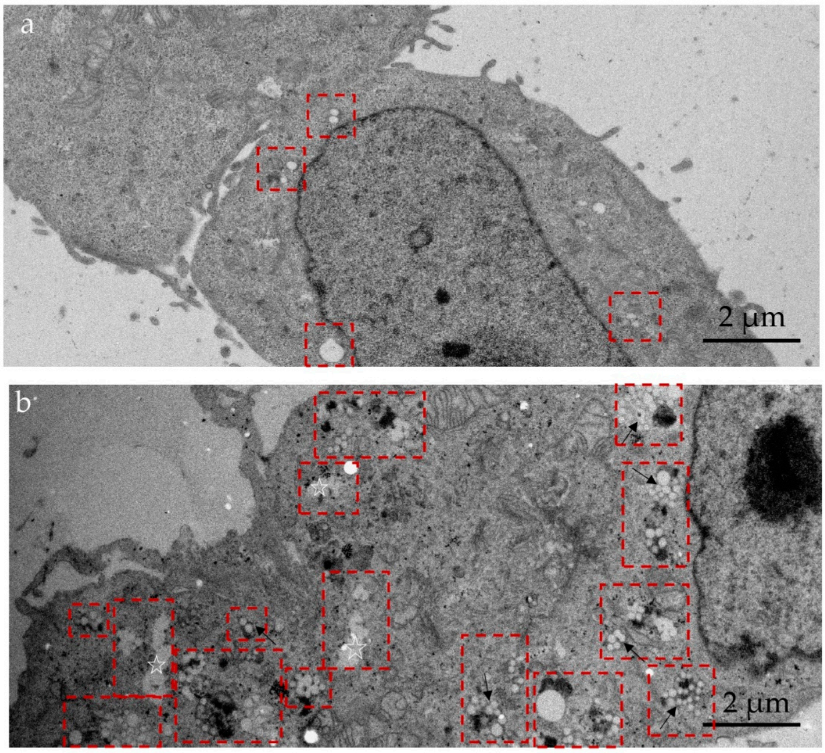

Figure S3. Differential uptake of NP T-B particles compared to NP in HeLa cells. Representative TEM images of HeLa cells treated with NP (**a**) or NP T-B (**b**) for 43 h. Red boxes indicate vesicles with internalized NPs. Black arrows indicate distinct NP T-B inside vesicles, while white stars denote internalized NP T-B at advanced stage of degradation (no visible limiting membrane between particles). Scale bar 2 μm.

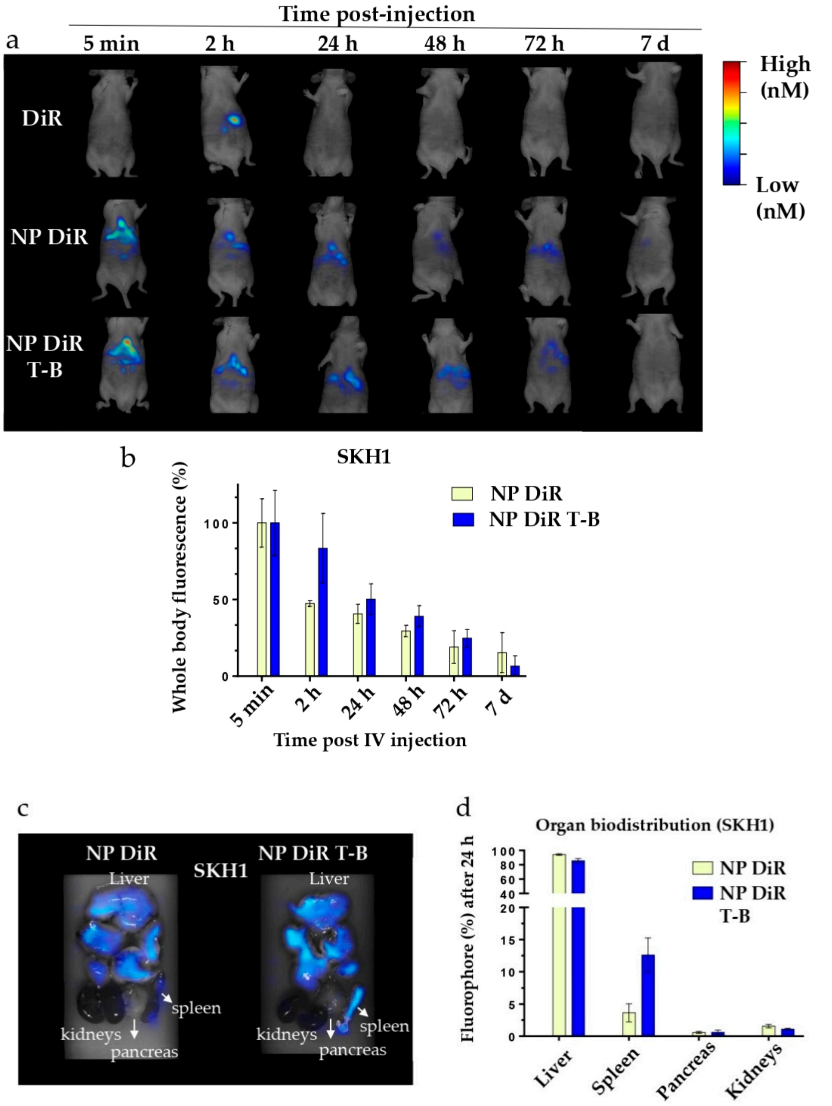

Figure S4. *In vivo* and organ DiR fluorescence imaging of SKH1 mice following a single IV injection (120 μL) of soluble fluorophore or fluorescent particles (0.25% w/v). (**a**) Autophagy inducing fluorescent particles (NP DiR T-B) and plain fluorescent particles (NP DiR) accumulate in the liver over the course of seven days. Scans are representative of three-four mice per group. (**b**) Quantification of whole-body fluorescence up to 7 days post a single IV injection. The percentage values reported are calculated from the 5 min timepoint, when the maximal fluorescence was observed, and are reported as mean ± s.e.m. Results were analyzed using one-way ANOVA followed by Sidak's multiple comparison test, which revealed no significant difference between the NP DiR and the NP DiR T-B group for all timepoints. (**c**, **d**) Organ biodistribution of fluorescent NPs 24 h after a single intravenous (IV) injection in SKH1. *n*=3-4 mice per group. *Ex vivo* DiR fluorescence imaging on main organs (liver, spleen, pancreas and kidneys) (**c**) and quantification of DiR fluorescence (**d**). The percentage values reported are calculated from total organ fluorescence and presented as mean ± s.e.m (**d**).

a

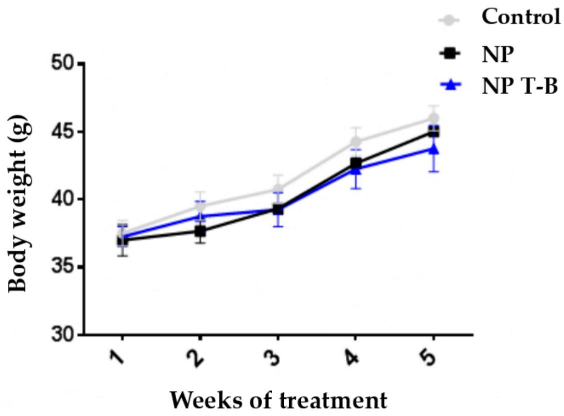

b

	ob/ob mice		
Group	Control	NP	NP T-B
Number of mice	4	3	4
Liver (g) to body (g) (%)	7.55 ± 0.20	7.04 ± 0.15	7.28 ± 0.39
**Liver function**			
ALT (UI/L)	292 ± 58	471 ± 191	400 ± 68
AST (UI/L)	356 ± 105	365 ± 107	390 ± 91

Figure S5. Effect of repeated administration of NP and NP T-B on body weight (**a**), liver to body ratio (**b**) and liver transaminases of ob/ob mice (**b**). Seven-week-old ob/ob mice were treated twice per week and for five consecutive weeks with 120 μL of buffer (Control), NP or NP T-B (n = 3-4 per group). No significant differences on body weight gain were observed at the end of the treatment period among the three treatment groups (two-way ANOVA and Tukey's multiple comparisons test (**a**) and no difference in the liver to body ratio and serum transaminases (one-way ANOVA and Tukey's multiple comparisons test) (**b**). Values in (**b**) are means ± s.e.m. ALT, alanine amino transferase; AST, aspartate amino transferase.
